# Examination of Poly (Styrene-Butadiene-Styrene)-Modified Asphalt Performance in Bonding Modified Aggregates Using Parallel Plates Method

**DOI:** 10.3390/polym11122100

**Published:** 2019-12-14

**Authors:** Xiangbing Gong, Zejiao Dong, Zhiyang Liu, Huanan Yu, Kaikai Hu

**Affiliations:** 1 State Engineering Laboratory of Highway Maintenance Technology, Changsha University of Science and Technology, Changsha 410114, China; huanan.yu@csust.edu.cn (H.Y.); kaikaihu@stu.csust.edu.cn (K.H.); 2 School of Transportation Science and Engineering, Harbin Institute of Technology, Harbin 150090, China; liuzhiyanghit@163.com

**Keywords:** rheology, microscopic characteristic, poly (styrene-butadiene-styrene)-modified asphalt, modified clamps, adhesion

## Abstract

Although asphalt-aggregate bonding provides contacting strength for hot mix asphalt (HMA), it is still ignorant in dynamic shear test, due to the only use of metal parallel plate. Modified parallel plates cored from different types of aggregate were provided to simulate aggregate-asphalt-aggregate (AAA) sandwich in HMA, aiming at the comprehensive interpretation on bonding’s influence. This study began with an experimental design, aggregate plates, and joint clamps were processed to be installed into the rheometer. Aggregate type and loading conditions were set as essential variables. Subsequently, microscopic tests were utilized to obtain chemical components of aggregate, micro morphology of interface, and roughness of plates. The shearing tests for poly (styrene-butadiene-styrene)-modified asphalt were conducted in bonding with aggregate plates. Meanwhile, contrasting groups adopting metal plates followed the same experimental procedures. The results indicate that the influence of aggregate type on binder’s rheological characteristics is dependent on the experimental variables, and microscopic characteristics and component differences should be taken into consideration when selecting aggregates in designing asphalt mixtures.

## 1. Introduction

Adhesive bonding [1] has been a major concern for many decades. The corresponding theory is being employed as a cutting-edge technique in the study for composite materials [2], and current research related to adhesion characteristics of asphalt-aggregate bonding has been taken account into hot mix asphalt (HMA). The corresponding methodologies can be divided into three major categorizations that are dependent on physical scales: micro-scale [3], meso-scale [4], and macro-scale [5]. Numerical simulations and analytical approaches are commonly applied at these scales, such as molecular dynamics (MD) [6], finite element method (FEM) [7], and discrete element method (DEM) [8]. A fact was known, as that asphalt-aggregate bonding illustrates an important impact on the performance of HMA. However, several assumptions contribute to be a trend analysis, rather than an accurate value for numerical methods.

Except for numerical methods, microscopic characteristics and mechanical tests were used to evaluate the impact of factors associated with the bonding interaction at the asphalt-aggregate bonding interface. Force-displacement curves that were provided by atomic force microscopy (AFM) were developed to obtain adhesive energy for thin asphalt films [9]. Instead of the silicon AFM tip, a calcium carbonate tip was modified to evaluate the bonding strength by simulating the microscopic interaction between asphalt and limestone [10]. Furthermore, a chemical model was provided to express a fundamental explanation of asphalt-aggregate bonding characteristics [11]. A former study found that the modulus of asphalt film fluctuated with the distance between asphalt and aggregate while using AFM [12]. Additionally, the size limitation of scanning at the micro-scale is insufficient for comprehensively understanding the impact of several factors at a larger scale, such as mineralogy [13], texture [13], and film thickness [14]. Currently, mechanical tests are generally conducted to exhibit adhering responses that were subjected to a given loading condition [15]. However, asphalt pavement is directly exposed to the environment, and temperature, moisture, and stress/strain are dominant factors. Thus, complicated filed conditions that are associated with multiple scales are challenges to completely understand the bonding interaction at the asphalt-aggregate interface.

The rheological characterization of asphalt is generally measured by a dynamic shear rheometer (DSR). Complex modulus (|G*|), phase angle (*δ*), and viscosity (*η*) are the typical outputs related to performance evaluation for binder [16], which are widely used to predict the stability [17] and the durability of polymer [18]. The variables for dynamic shear test consist of sample geometry, temperature, stress or strain level [19], and loading duration [20]. Thus, an appropriate experimental design is essential prior to the material preparation in this study. Moreover, instead of metal parallel plates, rock parallel plates were cored according to the protocols that were described in the Superpave binder specification. Several similar experimental designs have been provided to evaluate the asphalt-aggregate bonding behavior under various loading conditions, such as aging [21], changing film thicknesses [22], and moisture damage [23]. The rock plates were applied to test the shearing properties of asphalt [24] and asphalt mastic effectively [25], and aggregate-mastic interaction was finally illustrated based on a modulus model [26]. However, the obvious issue for those studies is that only one plate size was adopted, which limits the temperature range and loading form for the dynamic shear test.

**Figure 1:**
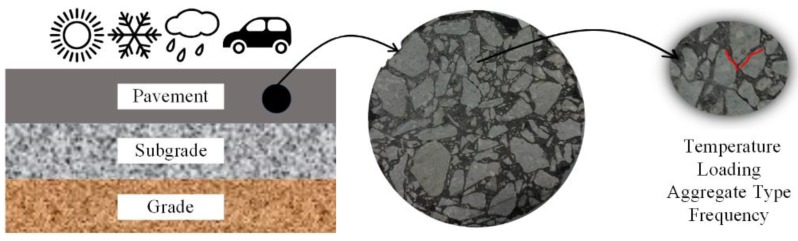
Schematic image to illustrate the purpose of aggregate-asphalt-aggregate dynamic shear rheometer (DSR) tests.

The primary motivation of this study is to design rock cylinders that can cover a wider temperature range based on a rectangle clamp of DHR-II (Discovery Hybrid Rheometer, TA Instruments, Lindon, UT, USA). One significant advantage for DHR-II is that the air bath has less interference rather than the water bath. An *Φ* 25 mm rock cylinder and an *Φ* 8 mm rock cylinder were both included in the experimental design. Therefore, the rheological properties of asphalt could be characterized at high, intermediate, and low temperatures that were subjected to different types of modified parallel plates. Another motivation is to conduct several tests by changing the variables to simulate the binder layer in real asphalt pavement (Figure 1), such as temperature, loading, aggregate type, and frequency. It aims at explaining how those fundamental variables affect the rheological characteristics of aggregate-asphalt-aggregate (AAA) system. This setup is designed to simulate the real conditions in asphalt pavement engineering partially. In addition, this study tries to provide a preliminary protocol to evaluate the influence of the aggregate plates on binder performances, and it will be improved based on the future works.

## 2. Materials and Experimental Design

### 2.1. Materials

Strategic Highway Research Program (SHRP) developed Superpave PG (performance gradate) asphalt binder specification. It defines a series of standard experiments that characterize the viscoelastic behavior of binder at multiple temperatures [27]. In this specification, the *Φ* 8 mm parallel plate is selected at intermedium temperatures, even low temperatures applying to a sample with 2000 μm thickness, *Φ* 25 mm plate is the corresponding clamps for oscillation experiments at high temperatures applying to a sample with 1000 μm thickness. Two types of rock were cored into *Φ* 8 mm and *Φ* 25 mm cylinders with a thickness of 5 mm. Two types of aggregate plate (limestone and basalt) were chosen, because they are used as the universal aggregate type in China. While the asphalt type was not designed to be a variable in this study, it is significant to select poly (Styrene-Butadiene-Styrene, SBS)-modified asphalt, because it is widely used. Based on several physical properties tests, the testing results are shown, as follows: penetration value is 58.3 (0.1 mm, 100 g, and 25 °C), softening point is 56.0 °C (ball and ring) while using the ring and ball method, and ductility is 40.1 mm (25 °C). Its fundamental properties meet the specification requirements of China.

### 2.2. Metal Base and Rock Cylinder Design

**Figure 2:**
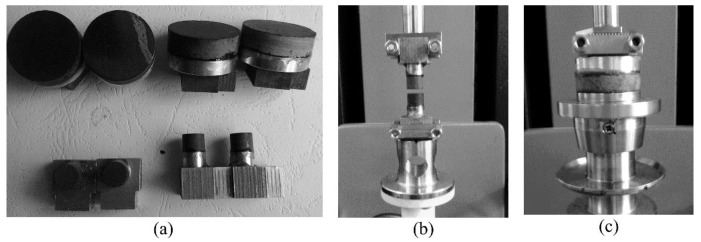
Experimental procedures: (**a**) rock cylinders (*Φ* 8 mm and *Φ* 25 mm) and bases after the glue operation, (**b**) installation in the Discovery Hybrid Rheometer (DHR) for the *Φ* 8 mm cylinder, and (**c**) installation in the DHR for the *Φ* 25 mm cylinder.

The first step was to process a base that is used as a joint part to connect a rock cylinder and a rectangle clamp of DHR. The base is divided into two parts: one part is an upper cylinder and other part is a lower polygon. The rock cylinder was fixed onto the upper part through the super glue [24]; the polygon bottom was fastened into the rectangle clamp of DHR through bolts and shims (Figure 2). A key procession was to ensure that the cylinder top should be concentric with the loading top of DHR, and thus several precise procedures were presented to generate the metal base. Ultimately, modified clamps were designed to have the same axis of symmetry with the loading system of DHR (Figure 2), which avoids eccentric force.

The next step was to core the rock cylinder into different diameters (8 mm and 25 mm), and two types of aggregate (limestone and basalt) were selected. Two types of upper cylinder were designed to be with 8 mm or 25 mm diameter, correspondingly. The rock cylinders were cored from aggregate slabs with a 5 mm height, and those cubic slabs were initially polished to create the parallel surfaces with a height tolerance of 0.02 mm [24]. However, there is no further polishing to reach a smooth surface, thus the microscopic roughness is still kept as the variable. Furthermore, the microscopic roughness of the rock cylinders was inspected by AFM and the chemical components were identified by X-ray fluorescence (XRF) and X-ray diffraction (XRD). Finally, gel epoxy was utilized to fix the rock cylinders onto the upper cylinder of the base. Super glue must be effective at temperatures ranging from −30 °C to 250 °C.

### 2.3. Experimental Description

Instead of metal plates, a pair of modified clamps can simulate an asphalt film that is sandwiched between two pieces of aggregate in HMA. It is essential to conduct the glue operation in the zero-gapped pattern [24]. Consequently, the upper and lower aggregate plate can be easily assembled to be concentric. When the epoxy is soft, the rock cylinders must be adjusted to be concentric with the upper cylinder. After 12 hours’ curing time, the modified parallel plates can then be installed into the rectangle clamps of DHR (Figure 2).

DHR is widely applied in the rheological research for poly-modified asphalt. Precise displacement/force control is the biggest advantage for DHR. The zero-gapping and trimming operations follow the same protocol that was described in the Superpave specifications, except for an additional curing process to generate the adhesive strength for the asphalt-aggregate interface (60 °C, 10 min). The aggregate plates must be heated to approximate 160 °C in an oven prior to the curing process and binder film installation. The function of this step is to dry out moisture and simulate the preheating process while blending for HMA.

In this study, the first test is the oscillatory sweep at a wide temperature range by adopting *Φ* 8 mm and *Φ* 25 mm plates, the second test is the relaxation test, and the last one is the multiple stress creep recovery test (MSCR). Each test has several groups: (a) three types of parallel plates, called basalt (BS), limestone (LS), and metal, respectively; (b) three pairs of parallel plates, named aggregate-aggregate (AA), aggregate-metal (AM), and metal-metal (MM), respectively; and, (c) several kinds of loading form, plate type, and frequency.

## 3. Results and Discussion

All of the tests were conducted within the upper limitation of LVE (Linear Viscoelasticity) range, and DHR was applied to measure the rheological outputs of asphalt film under different conditions. Complex modulus, relaxation modulus, and creep-recovery responses were the main outputs in this study. Afterwards, major results and discussion are described, as follows.

### 3.1. Microscopic Characteristics

#### 3.1.1. Morphology Analysis

The microscopic characteristics of aggregate surfaces cannot be ignored with respect to the asphalt-aggregate interface; SEM (Scanning Electron Microscope) and AFM are the effective observation methods [28]. SEM was used to investigate the morphology of asphalt-aggregate interface, and AFM determined the microscopic roughness of the cutting surface of aggregate.

**Figure 3:**
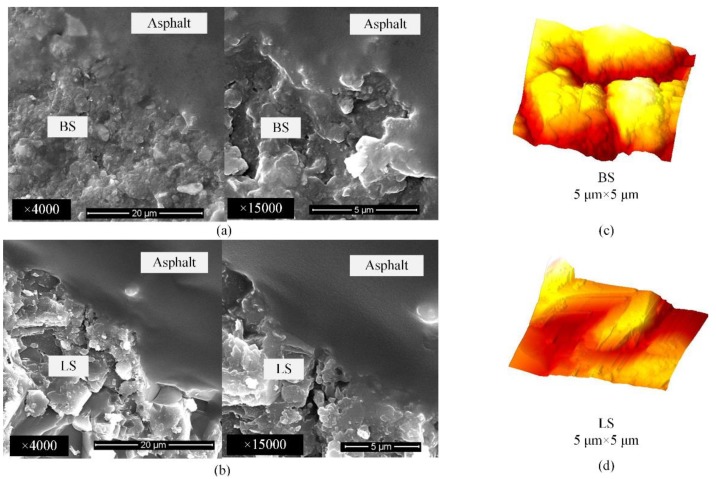
Microscopic characteristics: (**a**) Scanning Electron Microscope (SEM) images of basalt (BS), (**b**) SEM images of limestone (LS), (**c**) atomic force microscopy (AFM) image of BS, and (**d**) AFM images of LS.

Images that were taken by SEM and AFM in Figure 3 show conditions of the asphalt-aggregate bonding boundary at micro-scale. The samples were prepared by following the same procedures adopted in the experimental design in order to control the variable. Additionally, the average roughness (*R*_a_) of plates was calculated via *NanoScope Analysis Software,* which is known as a specific tool for AFM. In this paper, it mainly aims at micro roughness, except for macro shape of aggregate. Consequently, the aggregate plate surface was polished under the same polishing conditions. It is concluded that DHR metal plate (*R*_a_, 7.5 nm) has the smallest average roughness, followed by limestone (*R*_a_, 206.4 nm), and the largest one is basalt (*R*_a_, 264.8 nm). Micro roughness differences should be directly related to mineral components of various plates. The AFM phase images differentiate the surface morphology for BS and LS at micro-scale. Figure 3c exhibits that the valley is narrow and the peak is granular for the rough surface of BS. On the contrary, the valley is broad and the peak is flat for LS, as shown in Figure 3d.

Moreover, those differences can also be depicted from the SEM images. The microscopic defects in basalt is bigger than limestone, which could be clearly seen in Figure 3a,b. Therefore, hot asphalt can flow into the voids and easily fill the defects for basalt. Consequently, the boundary along the asphalt-basalt interface in Figure 3a was coated well. However, the boundary of asphalt-limestone interface in Figure 3b seems like an accumulation of binder without flowing into defects. The SEM images exhibit that the particles in limestone appear to be large cubic, which should block the flowing of hot asphalt. On the contrary, hot asphalt might coat those flat particles in basalt well. Therefore, it is concluded that basalt shows a better coating at the micro-scale due to the better distribution of microscopic morphology.

#### 3.1.2. Component Analysis

Not only microscopic morphology, but also the chemical component, are decisive factors that affect the microscopic interaction, because different chemical minerals must have a significant impact on absorbed asphalt proportion. In this part, the main objective is to differentiate mineral components. Besides, chemical component analysis can be used to check that the type of aggregate is correct. Consequently, XRF (X-Ray Fluorescence) and XRD (X-Ray Diffraction) were utilized to investigate chemical components of aggregate. XRF first determined the chemical elements of aggregate, and XRD was then applied into the elementary analysis of chemical components. For basalt, silicon takes the largest proportion, followed by iron and calcium. For limestone, silicon and calcium are the primary elements, followed by aluminum.

On the basis of the XRD results, the main compositions in aggregates were achieved. Calcite and quartz are the primary minerals for the limestone, which are mainly composed of CaCO_3_ and SiO_2_. Diopside, nepheline, and forsterite are the primary minerals for the basalt, being known as typical silicates. Based on the contact angle test [29], limestone has a smaller angle than basalt with respect to asphalt. A former study described that limestone exhibited a greater adsorption proportion of asphalt than quartzite and granite [30]. Additionally, numerical studies conclude that the limestone-asphalt samples have a better adhesive capability after boiling in the water bath. Therefore, it is estimated that limestone has the stronger adhesive capability that is associated with asphalt than basalt while accounting for chemical component differences.

### 3.2. Shearing Testing Results

Aiming at comprehensively evaluating the factors mentioned above, experimental plans focus on a wide temperature range that covers high, intermediate, and low temperatures. The outputs that were obtained through strain sweep, frequency sweep, relaxation test, and MSCR test are discussed, as follows.

#### 3.2.1. Strain Sweep

**Figure 4:**
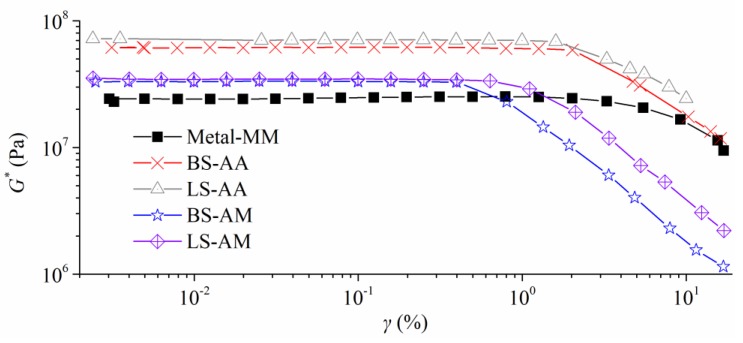
Strain sweep curves at 0 °C with different parallel plates (2000 μm, gap).

The low temperature cracking is a predominant issue that results in several early distresses of asphalt pavement. As the temperature drops, the pavement shrinks, then the aggregate-asphalt bonding zone tends to easily fracture because of the increasing tensile stress. In this part, the strain sweep experiment attempts to investigate the influence of strain on |G*| at a low temperature undergoing different bonding conditions. In addition, the test temperature was 0 °C with a constant frequency of 10 rad/s; Figure 4 shows the results.

The initial |G*| shows significant differences especially for different plate types. The AA plates have the greatest modulus, and followed by the AM plate, the MM plate has the smallest value. As strain increases, |G*|, as obtained from the AM plate significantly decreases, and followed by the AA plates and the MM plate. The largest decreasing percentage is clearly found in the AM plate, the MM plate shows the smallest reduction rate. Additionally, the LS plate generates the better resistance to the increasing strain than the BS plate at 0 °C, no matter what pair type is. These findings indicate that limestone-asphalt-limestone system is the strongest interaction among those plates at 0 °C. However, the |G*| that was measured from aggregate plates are easier to decrease with the increase of strain than metal plates. This finding implies that smooth aggregate surface could be concluded as an advantage when asphalt pavement is located in the cold region.

#### 3.2.2. Frequency Sweep Using *Φ* 8 mm Plates

**Figure 5:**
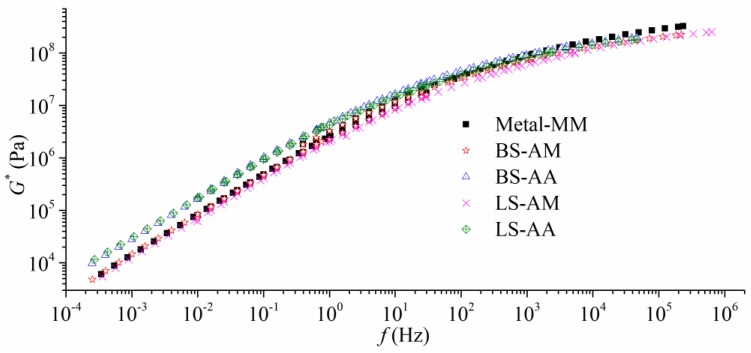
Master curves at 15 °C with different types of aggregates and plates (2000 μm, gap).

Modified 8 mm parallel plates were used to obtain master curves at intermediate temperatures and low temperatures. The shearing modulus was measured by DHR with various frequencies ranging from 0.01 Hz to 30 Hz, and the tests were conducted at four temperatures (−15 °C, 0 °C, 15 °C, and 30 °C). Figure 5 shows the master curves of modulus at 15 °C, and the results and discussion are presented, as follows.

It is clear that the AA plates contribute to the largest modulus with respect to low-frequency shearing. While, the master curve of the MM plate moves upward in Figure 5 as the frequency increases via contrasting with the AA plate and the AM plate. However, the modulus curves for the AA and AM plates tend to be smaller than the MM plate as the frequency increases. The modulus differences between the AA plates and the MM plate become larger with the increase of gap. It is summarized that |G*| for the MM plate is nearly the largest when the frequency is around 10^4^ Hz. It could be concluded that smooth surface might be a good property for the AAA system associated with high-frequency loading, as well as low-frequency loading at intermediate temperature.

Table 1 and Figure 5 present a comparison between the AM plate and the MM plate. The modulus between the AM plate and MM plate are close when the frequency is low. As the frequency gets higher, |G*| for the AM plate becomes smaller than the MM plates. In Table 1, SBS binder modulus are significantly affected by the type of aggregate verified by experimental results from the BS and LS groups, and the basalt generate a stiffer AAA system than limestone at an intermediate temperature. This finding demonstrates that aggregate chemical components should be treated as factors when designing intermediate temperature properties of mixtures, such as fatigue.

#### 3.2.3. Frequency Sweep Using *Φ* 25 mm Plates

**Figure 6:**
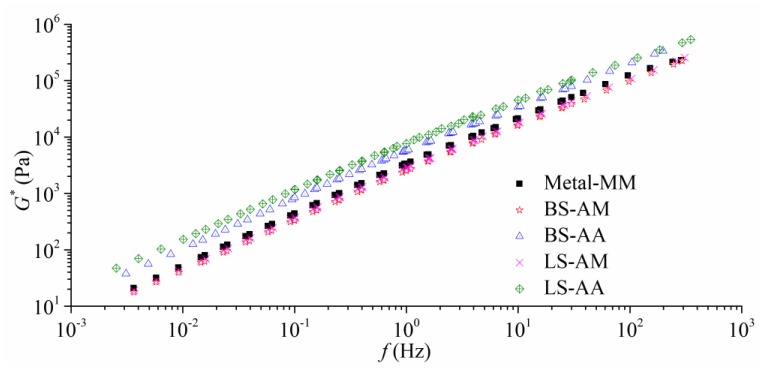
Master curves at 60 °C with different types of aggregates and plates (1000 μm, gap).

Modified *Φ* 25 mm plates were employed into frequency sweep at high temperatures. In this section, the frequency sweep tests were generally conducted at four temperatures (45 °C, 60 °C, 75 °C, and 90 °C). The dynamic frequency changed from 0.1 Hz to 30 Hz. Figure 6 illustrates the aster curves at 60 °C; the results can be concluded, as follows.

All of the solid plates are less sensitive to the frequency when comparing Figure 6 with Figure 5. As seen from Figure 6 and Table 2, |G*| for the AA plates is greater than the MM plates, and the smallest |G*| is found in AM plates. Therefore, the homogeneous aggregate property is also an index in selecting aggregates that are subjected to high temperatures. The largest |G*| for the AA plates and AM plates are both found in limestone. In Figure 3, the microscopic roughness of limestone is not the greatest one, but the well-coating boundary might result in the largest |G*| at high temperature. Consequently, the strong bonding could predominate the shearing flow behavior of AAA system, being associated with the high-temperature stability of HMA.

#### 3.2.4. Relaxation Test

**Figure 7:**
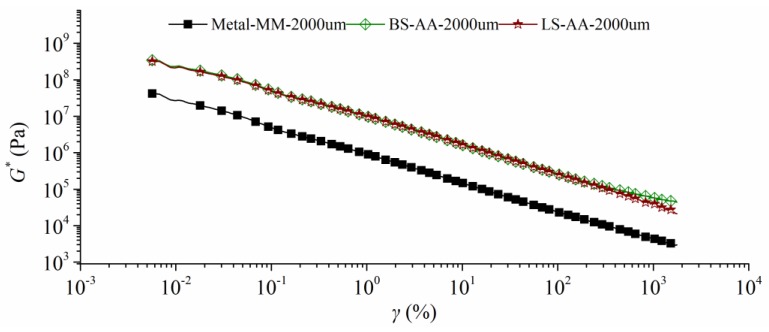
Relaxation modulus curves at 0 °C with different parallel plates: metal-metal (MM) plates, basalt-aggregate-aggregate (BS-AA) plates, and limestone-AA (LS-AA) plates (2000 μm, gap).

The relaxation phenomenon is defined as modulus decreases undergoing a constant strain. It is a typical factor that is related to the resistance to the cracking of asphalt pavement at low temperatures. A rapid decrease of relaxation modulus (*G*(*t*)) refers to the strong resistance to the thermal cracking. The relaxation test of AAA system was conducted under strain mode, based on the modified parallel plates. The experimental temperature was 0 °C, and the shear strain was kept as a constant (1%). *G*(*t*) versus time data was measured by DHR for the AA and the MM plates, Figure 7 presents the findings depicted, as below.

In Figure 7, the initial SBS binder stiffness is related to the plate type. The smooth metal plate leads to the smallest initial *G*(*t*), which means that the thermal stress would be reduced in the metal-binder-metal system. Although the *G*(*t*) curves of the BS-AA plate and the LS-AA plate seem to be similar, the curve of the BS-AA plate tends to be flat when comparing with the LS-AA plate and MM plate. Thus, it could be summarized that the smooth surface results in easy thermal stress relaxation. This observation denotes that rough aggregate surface at the micro-scale could degrade the resistance to thermal cracking of asphalt mixture, especially suffering a long-term cold climate or a sharp temperature dropping climate.

#### 3.2.5. MSCR Test

Instead of the rutting factor, the multiple stress creep recovery test (MSCR) is widely used to amend the evaluation index at high temperatures for poly-modified asphalt [31]. This test generally covers two stresses (100 Pa, 3200 Pa), and each test repeats ten cycles. Each cycle endures one second creep loading and then recovers for nine seconds without loading. The creep strain at the end of one second’ loading is StrainA*_i_*, the recoverable strain at the end of nine seconds’ recovery is StrainB*_i_*. Namely, the non-recoverable strain (StrainC*_i_*) is the difference between StrainA*_i_* and StrainB*_i_*. The recoverable rate (*R*, %) and non-recoverable creep compliance (*J*_nr_, kPa^−1^) are effective parameters for analyze results [32].

**Figure 8:**
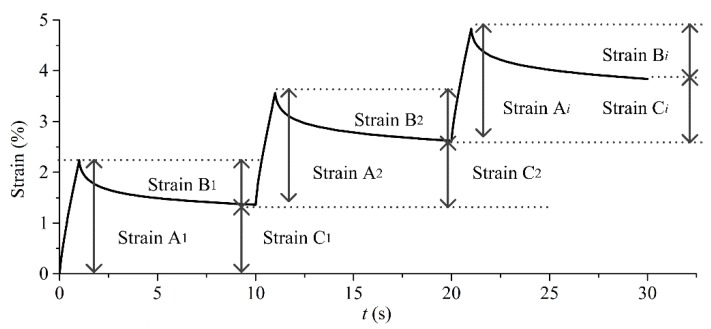
Strain illustration for the multiple stress creep recovery test (MSCR) test and the initial strain is assumed to be zero for the first cycle.

**Figure 9:**
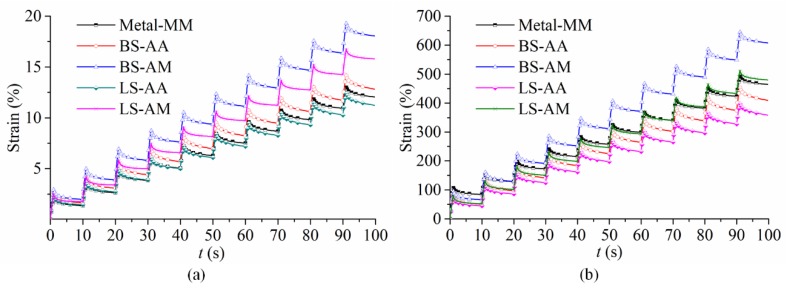
Strain versus time plots of different types of aggregates and plates (1000 μm, gap): (**a**) 100 Pa, and (**b**) 3200 Pa.

**Figure 10:**
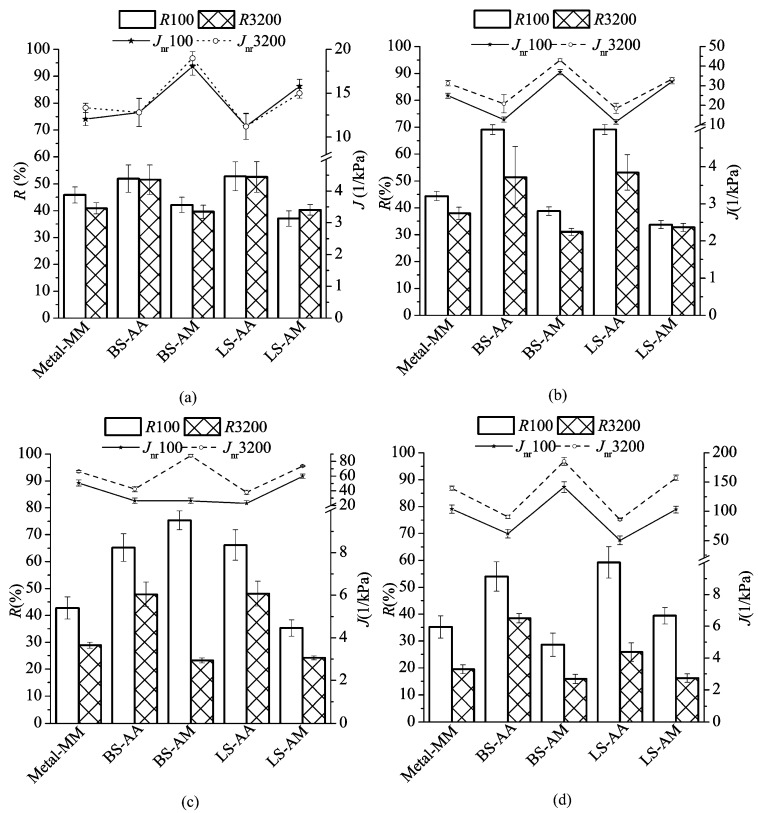
Two parameters calculated from the MSCR test: (**a**) 45 °C, (**b**) 50 °C, (**c**) 55 °C, and (**d**) 60 °C, the error bar is equal to the standard deviation, 100 means 100 Pa, and 3200 represents 3200 Pa.

According to the strain illustration (Figure 8), all of the parameters could be calculated via formulas exhibited as below, and *i* means the cycle number. The MSCR test for SBS-modified asphalt utilizing different plates was conducted at four temperatures (45 °C, 50 °C, 55 °C, and 60 °C) with a gap of 1000 μm. The strain versus time was recorded by DHR, and strain can be drawn and found in Figure 9. In Figure 10, the bars represent *R* and the plots represent *J*_nr_, the results and discussion are shown, as follows.

(1) $$R100=\left(1/10\right)\left\{\overset{10}{\underset{i=1}{\sum }}{\mathrm{StrainB}}_{i}/{\mathrm{StrainA}}_{i}\right\}*100$$

(2) $$R3200=\left(1/10\right)\left\{\overset{10}{\underset{i=1}{\sum }}{\mathrm{StrainB}}_{i}/{\mathrm{StrainA}}_{i}\right\}*100$$

(3) $$J_{\mathrm{nr}}100=\left(1/10\right)\left\{\overset{10}{\underset{i=1}{\sum }}{\mathrm{StrainC}}_{i}/0.1\right\}$$

(4) $$J_{\mathrm{nr}}3200=\left(1/10\right)\left\{\overset{10}{\underset{i=1}{\sum }}{\mathrm{StrainC}}_{i}/3.2\right\}$$

It can be found that the *J*_nr_ increases as the temperature goes up no matter what kind of plate and stress were applied. In Figure 10a, the curves of *J*_nr_ for different stress are similar. However, the differences between *J*_nr_100 and *J*_nr_3200 present an undeniable increase when the temperature changes from 45 °C to 60 °C, and the greatest gap is approximately 60 kPa^−1^. The AA plate has a smaller *J*_nr_ than others, and the AA plate with limestone delivers a better recoverable performance than the basalt. The AM plate with basalt exhibits the highest *J*_nr_ while excluding the *J*_nr_100 at 55 °C, which indicates that the microscopic roughness could be a critical factor affecting the recoverable resistance of the AAA system in HMA at high temperatures.

Accounting for *R* bars, one finding is that value gap between *R*100 and *R*3200 becomes larger with the increase of temperature. It is summarized like that a high temperature combined with a heavy traffic would significantly degrade the displacement recoverable capability of the asphalt mixture. Another finding can be concluded as that the AA plate shows the best resistance to the creep deformation. However, the *R*100 at 55 °C for the AM plate with basalt rises from 37% to 75%, the gap ranks as the highest increasing rate. This observation might be related to a better binder coating at the micro-scale for the basalt-asphalt interface zone that is shown in Figure 3.

The *R*3200 decreases as the temperature increases, but *R*100 presents more complicated fluctuation when it comes to the AA plate. First, the *R*100 of two types of plate increases to around 70% at 50 °C. Subsequently, it decreases by approximate 5% at 55 °C. Finally, the *R*100 of the AA plate with basalt and limestone reduces to 54% and 60%. The microscopic roughness could be a major index that results in the fluctuation of *R,* just when the shearing loading is not too heavy. At the highest temperature of 60 °C, the maximum *R*100 occurs in the AA plate with limestone, and the maximum *R*3200 occurs in the AA plate with basalt, which might be owed to its microscopic roughness. When considering the rutting issue at high temperatures, limestone would be a good choice while undergoing regular traffic, but the basalt should be the preferred aggregate type subjected to a heavy traffic.

## 4. Conclusions

This study aims at understanding how different aggregate-modified plates affect the rheological characteristics of poly (SBS)-modified asphalt, and a development of this study is to conduct modified shearing tests at a wide temperature range as compared with former studies. Several conclusions can be summarized based on the analysis mentioned above.

Aggregate selection in the asphalt mixture design is a dominant procedure for generating a strong asphalt mixture. Additionally, the aggregate-plate-modified DSR testes developed in this study could provide instructions to choose an optimum aggregate type. The testing methods would be further used to predict HMA performances under various conditions, such as different temperatures and traffic volumes.

One obvious fact is that the MM plate is less sensitive to DSR testes than other plates, which lacks the capability to illustrate AAA properties in HMA. Aggregate-modified DSR tests are effective in investigating the influence of AAA bonding and simulate a real AAA system in asphalt mixtures, which involves binder coating at the micro-scale and aggregate chemical components.

The effects of microscopic roughness and chemical components are dependent on temperature, frequency, and loading form. Rough aggregates combined with strong adhesive capability could improve the asphalt mixture performances. In this study, similarity among aggregate particles is also known as a critical index, because it might contribute to homogeneous interaction within the AAA system. Therefore, the source of stone must be seriously controlled to avoid large property variability.

## 5. Future Research

More aggregate types and asphalt types will be included in future works. Additionally, the macro shape of an aggregate needs to be involved to interpret the shearing behavior of the AAA system. Next, the experimental design will focus on the thickness effect as well as the compliance verification of DSR testing subjected to difference film thinness.

## Figures and Tables

**Table 1:** Dynamic modulus at 15 °C with different frequencies and plates (2000 μm, gap).

Frequency (Hz)	Dynamic Modulus (Pa)				
	Metal-MM	BS-AM	BS-AA	LS-AM	LS-AA
0.01	8.04 × 10^4^	7.21 × 10^4^	1.72 × 10^5^	6.27 × 10^4^	1.74 × 10^5^
1	2.40 × 10^6^	2.24 × 10^6^	4.61 × 10^6^	2.06 × 10^6^	4.21 × 10^6^
10	9.86 × 10^6^	9.00 × 10^6^	1.65 × 10^7^	8.27 × 10^6^	1.51 × 10^7^
30	1.74 × 10^7^	1.59 × 10^7^	2.73 × 10^7^	1.44 × 10^7^	2.52 × 10^7^

**Table 2:** Dynamic modulus at 60 °C with different frequencies and plates (1000 μm, gap).

Frequency (Hz)	Dynamic Modulus (Pa)				
	Metal-MM	BS-AM	BS-AA	LS-AM	LS-AA
0.01	4.35 × 10^2^	3.31 × 10^2^	8.42 × 10^2^	3.64 × 10^2^	1.17 × 10^3^
1	3.34 × 10^3^	2.55 × 10^3^	5.68 × 10^3^	2.82 × 10^3^	7.66 × 10^3^
10	2.14 × 10^4^	1.63 × 10^4^	3.41 × 10^4^	1.80 × 10^4^	4.51 × 10^4^
30	5.07 × 10^4^	3.87 × 10^4^	7.95 × 10^4^	4.29 × 10^4^	1.02 × 10^5^
